# Commentary: Deep Phosphoproteomic Measurements Pinpointing Drug Induced Protective Mechanisms in Neuronal Cells

**DOI:** 10.3389/fphys.2017.00174

**Published:** 2017-03-21

**Authors:** Amit K. Yadav

**Affiliations:** Drug Discovery Research Center, Translational Health Science and Technology Institute, NCR Biotech Science ClusterHaryana, India

**Keywords:** phosphoproteomics, Alzheimer disease, quantitative proteomics, posttranslational modifications, neurological disorders

Cellular signaling controls an organism's molecular milieu in two ways—by facilitating internal growth and development, as well as responding to external stimuli, which includes response to environmental stress and drug effects. Signaling networks control the information flow at molecular level in a robust manner despite some inherent noise, tightly controlling the molecular determinants of homeostatic balance. Several drug candidates are under trial for neurodegenerative disorders for which molecular mechanism is not known in detail. Most drugs act on more targets (including off-targets) than previously thought. Studying the mechanisms of drug action is thus important not only for understanding biology, but also for design of more potent therapeutic molecules. If the molecular underpinnings are adequately understood, those can be easily tinkered with without causing toxicity or adverse effects. Exploration of mechanisms of drug action by studying drug-induced signaling perturbations using quantitative proteomics is gaining momentum (Sacco et al., 2016).

Yu and coworkers recently studied the phosphoproteomic changes in neuro-2a cells challenged by cytotoxicity from Aβ_25−35_ peptide and the reversal of the changes by drug candidate- GFKP-19, a 2-pyrrolidone derivative compound (Yu et al., 2016). Using SILAC labels, three different treatment conditions were studied in neuro-2a cells- light (DMSO control), medium (cells with Aβ_25−35_) and heavy (Aβ_25−35_ + GFKP-19) on Q-Exactive (QE) and Q-Exactive HF (HF) mass spectrometers. Specific phosphorylation changes were captured with ~25,000 phosphorylation sites of which ~18,000 have high localization probability. The important regulatory perturbations in phosphoproteome studies at 4 and 24-h in each condition were captured and as expected, the authors found that the 4-h time period shows more regulation of the phosphoproteome than 24-h, since the signaling changes in phosphoproteins will be driving the information flow in early time period. Regulated phosphosites between control vs. Aβ-treatment and drug vs. Aβ were observed to control cytoskeletal protein functions. Authors also found Tau phosphorylations, which are known causative agent of aggregation in AD. Proline directed kinase (PDK) motifs were found in motif analysis along with basophilic motifs in both Aβ treatment and the drug rescue conditions. Oxidative damage due to aggregation is caused by superoxide radicals binding to NO which can be formed by nitric oxide synthases (NOS1, NOS2, or NOS3). Aβ induces phosphorylation of NOS1 at S847 which is reversed by GFKP-19 bringing down oxidative damage. Another site S479 of ASPP2 increased with Aβ treatment was rescued by the drug but the functional aspect of this site is unknown. Six sites in Tau protein were upregulated out of 20, which had known counterparts in humans and the drug reversed the effect in all 6 sites. From the interaction network, authors postulated that phosphorylation of MARK2 and MAPK14 may be involved in AD based on several phosphosites although many previously known sites were not observed. For more depth in understanding, profiling of these molecular changes in phosphoproteome at a finely-resolved temporal level will be interesting, as literature has shown phosphoproteome level regulation starts much earlier than studied here. This may require a multiplexed quantitative proteomics technique like hyperplexing combining SILAC and iTRAQ (Kumar et al., 2016), or the label free SWATH. While SWATH is experimentally more challenging, hyperplexing is experimentally much easier, even though costlier in comparison. For validation and quantitation of PTM sites in a targeted manner, selected reaction monitoring (SRM) can be employed on a triple quadrupole mass spectrometer (Lange et al., 2008).

Role of Tau protein hyperphosphorylation is already well known in AD and is not a surprise that the authors chose to study Tau phosphosites in detail. Yet, it was interesting to find out newer phosphosites regulated in drug mediated protective mechanisms. It is also well accepted that all sites of phosphorylation may not be functional and transient non-specific phosphorylation sites may be captured in the experiments; a detailed site-mutational study may be warranted to check the functional significance of the mapped sites (Beltrao et al., 2012). While the study of phosphoproteome is deeply profiled and quantified, AD is a disease caused by dysfunction of microtubules assembly and function. O-GlcNAcylation is greatly reduced in AD leading to increased Tau hyper-phosphorylation. Tubulin assembly and maintenance is also arbitrated by several other PTMs and perhaps their dynamic crosstalk. Several modifications like detyrosination, acetylation, and polyglutamylation are known to govern assembly maintenance and function of α-tubulin and microtubules. Several tubulin modifications have a compensatory effect against microtubule destabilization in susceptible neurons in AD (Zhang et al., 2015). It will be interesting to see follow-up studies on coordinated action of phosphoproteome with other PTMs like acetylation and ubiquitination using serial enrichment (Mertins et al., 2013).

## Author contributions

The author confirms being the sole contributor of this work and approved it for publication.

## Funding

AKY is supported by IYBA grant from Department of Biotechnology (DBT), Ministry of Science and Technology, India.

### Conflict of interest statement

The author declares that the research was conducted in the absence of any commercial or financial relationships that could be construed as a potential conflict of interest.
